# Microporous methacrylated glycol chitosan-montmorillonite nanocomposite hydrogel for bone tissue engineering

**DOI:** 10.1038/s41467-019-11511-3

**Published:** 2019-08-06

**Authors:** Zhong-Kai Cui, Soyon Kim, Jessalyn J. Baljon, Benjamin M. Wu, Tara Aghaloo, Min Lee

**Affiliations:** 1 Department of Cell Biology, School of Basic Medical Sciences, Southern Medical University, Guangzhou, Guangdong 510515 China; 2 Division of Advanced Prosthodontics, University of California Los Angeles, 10833 Le Conte Avenue, Los Angeles, CA 90095 USA; 3 Department of Bioengineering, University of California Los Angeles, 420 Westwood Plaza, Los Angeles, CA 90095 USA; 4 Division of Diagnostic and Surgical Sciences, University of California Los Angeles, 10833 Le Conte Avenue, Los Angeles, CA 90095 USA

**Keywords:** Biomaterials, Tissue engineering, Biomaterials, Nanoscale materials

## Abstract

Injectable hydrogels can fill irregular defects and promote in situ tissue regrowth and regeneration. The ability of directing stem cell differentiation in a three-dimensional microenvironment for bone regeneration remains a challenge. In this study, we successfully nanoengineer an interconnected microporous networked photocrosslinkable chitosan in situ-forming hydrogel by introducing two-dimensional nanoclay particles with intercalation chemistry. The presence of the nanosilicates increases the Young’s modulus and stalls the degradation rate of the resulting hydrogels. We demonstrate that the reinforced hydrogels promote the proliferation as well as the attachment and induced the differentiation of encapsulated mesenchymal stem cells in vitro. Furthermore, we explore the effects of nanoengineered hydrogels in vivo with the critical-sized mouse calvarial defect model. Our results confirm that chitosan-montmorillonite hydrogels are able to recruit native cells and promote calvarial healing without delivery of additional therapeutic agents or stem cells, indicating their tissue engineering potential.

## Abstract (web summary)

Injectable hydrogels could be used to repair bone defects. Here the authors incorporate nanoclay particles into chitosan creating an interconnected microporous hydrogel and show that this hydrogel can support MSC proliferation and differentiation in vitro, and support the recruitment of native cells and bone regeneration in a mouse calvarial defect model.

## Introduction

Tissue engineering exploits a combination of cells, engineering and materials methods, along with proper biochemical and physiochemical factors to improve or replace biological tissues, including the skin, cartilage, bladder, blood vessels, and bone^1^. The decisive contribution of the tissue engineering matrix in ensuring the regeneration potential of stem and progenitor cells has been emphasized increasingly^2–4^. Progress in cell selection, cell culture, and new material formulations has led to the development of more effective therapies for tissue engineering and regenerative medicine (TERM). Various materials have been explored for regenerative applications, including naturally occurring products and synthetic materials^5,6^. Generally speaking, the intrinsic properties of naturally derived materials, such as collagens, are definitely attractive; however, the complexities of purification, immunogenicity, mechanical properties, and pathogen transmission significantly limit their application. In light of this, greater control over material properties and tissue responses has garnered more attention based on designable modern material science.

Biomaterials are having great impact on the medical treatments in helping to solve clinical problems. In the field of medicine, drug-eluting stent coated with polymers and controlled drug release systems constituted with biomaterials have been saving hundreds of thousands of lives each year^7,8^. In the field of TERM, the combination of biomaterial scaffold and certain cells is possible to replace biological tissues^1^. In the field of medical devices, biomaterials are also playing a significantly important role as the core components in surgical sutures, bioadhesives, and dental implants^9^. Clays and clay minerals are emerging materials for biomaterial design to provide new strategies for TERM. The usage of inorganic layered nanomaterials for medical purpose dates back to ancient time, such as for wound healing and hemorrhage inhibition^10^. Nowadays, clays and clay minerals are being applied in pharmaceuticals as active ingredients or excipients, and in cosmetics as creams, powders, and emulsions^10^. The interactions between clay nanoparticles and drugs as well as other biological molecules have been well investigated and therefore exploited for controlled delivery^11,12^, and moreover, the addition of clay into polymers enhances the mechanical properties owing to the formation of nanocomposites^13,14^.

Montmorillonite (MMT) is a major component of Bentonite, which is already approved by the FDA as an additive in various medicinal products^11^. Studies on the acute and chronic toxicities of MMT have confirmed the absence of any negative effects, even on embryos of pregnant Sprague–Dawley rats^15,16^. MMT is a layered silicate [(Na,Ca)_0.33_(Al,Mg)_2_Si_4_O_10_(OH)_2_•*n*H_2_O], belonging to the smectite group of minerals, with high specific surface area (up to 600 m^2^ g^−1^) and aspect ratio. The repeating structural unit of MMT consists of one alumina octahedral sheet sandwiched in between two silicon tetrahedral layers. The overall surface charge is light negative because of the domination of the oxide anions, which facilitates mixing with cationic agents. The MMT particles are typically in a plate-shape, rendering ~1 nm in thickness and 0.2–2 μm in diameter. The biocompatibility, availability, and feasibility of this particular mineral has garnered significant attention in recent years. Extensive research has been carried out to investigate for the purpose of drug and gene delivery with natural or modified MMT^11,17,18^. In addition, several reports have demonstrated that fabrication of scaffolds by introduction of MMT in natural biomaterials, including gelatin^19,20^, collagen^21^, silk^22^, and chitosan^23,24^, improved cell-scaffold interactions, cell proliferation, and enhanced cell differentiation. Although all those hard scaffolds were reported with porous structure under high vacuum and dry conditions, besides those experiments were limited only in vitro, which are not ideal to be applied in TERM, as it is not possible to include any live cells during fabrication of those scaffolds with the conventional methods to create porous structure, such as freeze-drying, porogen leaching. After the establishment of the scaffolds, cells were loaded leading to nonuniform distribution. Considering this, MMT is of interest for the formation of porous structure, and to be investigated further in vivo for TERM as an application.

Hydrogels derived from natural products are an appealing three-dimensional biomaterial for tissue engineering. Compared with hard scaffolds that require pre-shaping, soft hydrogels are injectable and can fill any irregular shaped defects in a minimally invasive manner. Recently, we have developed a photoinducible hydrogel system of methacrylated glycol chitosan (MeGC) using riboflavin as a photoinitiator^25^. MeGC hydrogels supported proliferation and extracellular matrix deposition of encapsulated mesenchymal stem cells^25,26^; however, the hydrogel itself showed minimal bone-forming ability without osteogenic factors, bioactive molecules, or encapsulated cells. One of the possible reasons is lacking of porous structure in the MeGC hydrogel. Porosity is reported to be necessary for new tissue formation as it allows the cells to migrate, infiltrate, and proliferate in a 3D environment, as well as for the vascularization, differentiation, and mass transport^27^. Therefore, it is of interest to develop a hydrogel system that could recruit native cells and facilitate bone formation with a microporous interconnected structure.

In this study, we introduce MMT to the photopolymerizable MeGC hydrogel system to fabricate an injectable highly osteoconductive in situ-forming biomaterial for bone regeneration. We hypothesize that MMT can not only improve the microstructure but also enhance the mechanical properties of cured MeGC hydrogel. Therefore, we started with optimizing the proportion of MMT in hydrogels and investigated the osteogenesis of encapsulated mesenchymal stem cells in vitro. We further evaluated the ability of the nanocomposite hydrogels to recruit native cells and improve bone formation in a critical-sized mouse calvarial defect model. The treatment of bone defects remains one of the largest challenges in musculoskeletal TERM, thereby our developed nanosilicate-loaded MeGC hydrogel may represent a new material design in the broadly interesting area of growth factor free and cell-free strategies. This bioactive nanocomposite hydrogel can provide an effective treatment for bone defects.

## Results

### Characterization of MeGC-MMT nanocomposite hydrogels

**Fig. 1:**
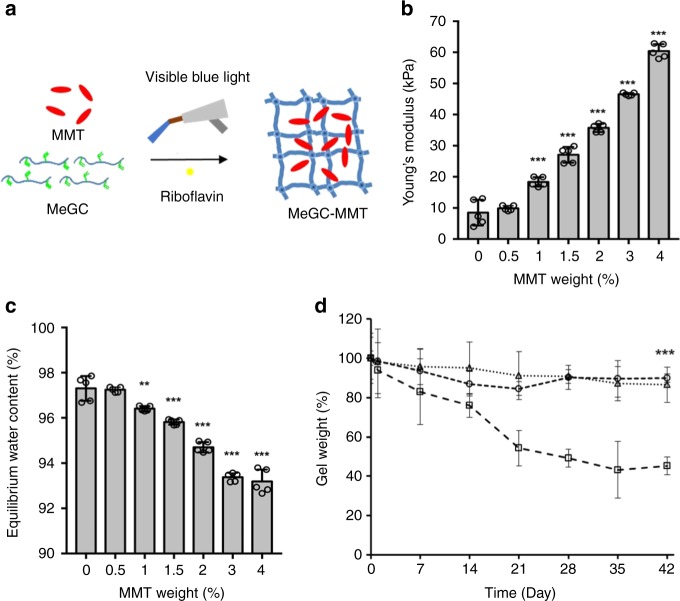
Characterization of photocross-linked MeGC-MMT nanocomposite hydrogels. **a** Schematic illustration of MeGC-MMT hydrogel cured by visible blue light cross-linking with riboflavin as the photoinitiator in the presence of MMT. **b** Young’s modulus of MeGC-MMT nanocomposite hydrogels with various amount of MMT. **c** Equilibrium water content of MeGC-MMT nanocomposite hydrogels. **d** Degradation profiles of MeGC-MMT nanocomposite hydrogels in the presence of 0% (square), 1.5% (circle), and 3% (triangle) MMT in PBS at 37 °C for 42 days. Error bars indicate standard deviation (*n* = 5). *p < 0.05, **p < 0.01, and ***p < 0.001 compared with the control MeGC group with 0% MMT (ANOVA followed by Tukey’s post hoc test)

Nanocomposite hydrogels including various amount of MMT (0.5-4% w/v) were prepared as illustrated in Fig. 1a. We already tested higher amount of MMT up to 10% in MeGC hydrogels. It became too viscous to mix with poor handling, as the MMT ratio increased over 4%. Therefore, we have selected the MMT ratio between 0.5 and 4% for the investigation. The electrostatic interactions between the heterogeneously distributed charges of discotic MMT (overall negative charge) and MeGC hydrogel matrices (positive charge) enhanced the Young’s modulus with an increasing amount of MMT in the nanocomposite hydrogels from 10 kPa to over 60 kPa (Fig. 1b). Equilibrium water content (EWC) inversely represents the cross-linking density and the mechanical properties of a hydrogel^28^. Introduction of more than 0.5% MMT in MeGC hydrogels significantly decreased the EWC of corresponding nanocomposite hydrogels (Fig. 1c), indicating enhanced mechanical properties. The dry weights of the MeGC-MMT nanocomposite hydrogels were recorded for 42 days (Fig. 1d). As observed in the degradation profiles, the degradation rate of the 1.5% and 3.0% MMT incorporated MeGC hydrogels was significantly decreased compared with the control MeGC hydrogels by ~40%. TGA degradation profiles confirmed the presence of MMT enhanced the thermal stability of MeGC hydrogels (Supplementary Fig. 1).

**Fig. 2:**
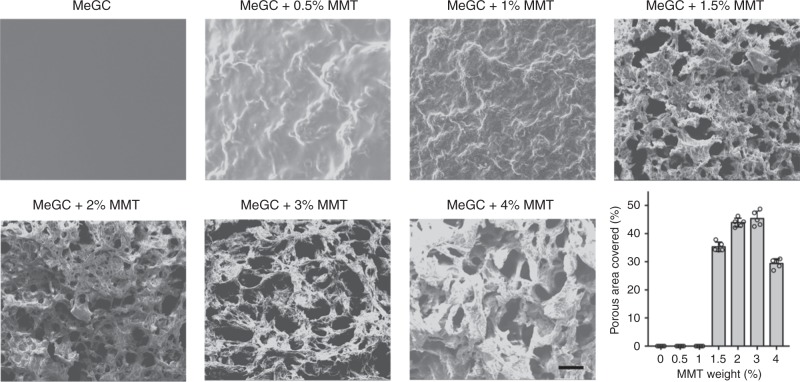
Characterization of interconnected microporous structure. SEM images of photocross-linked MeGC-MMT nanocomposite hydrogels. Various amount of MMT ranged from 0 to 4% (scale bar = 100 μm). Percentage porous area covered was quantified by SEM image analysis. Error bars indicate standard deviation

The microstructure of MeGC and MeGC-MMT hydrogels observed by SEM (Fig. 2) confirms the formation of a microporous and interconnected network when MMT exceeds 1.5%, while a smooth continuous surface was acquired for the control MeGC group, and a coarse continuous surface appeared for 0.5 and 1% MMT groups. Porosity was quantified as an index of surface area occupied by the pores in the SEM images. The pore sizes were around 115 ± 40 μm for the 1.5% MMT group. With the increasing MMT content, the pore sizes reached the maximum of 150 ± 50 μm for the 3.0% MMT group. The involvement of MMT thoroughly changed the microstructure of MeGC hydrogels. In addition, MMT was well distributed in the MeGC hydrogel matrices in all the nanocomposite groups without aggregation, which was confirmed by EDS (Supplementary Table 1) and TEM observation (Supplementary Fig. 2).

### Cytocompatibility of MeGC-MMT nanocomposite hydrogels

**Fig. 3:**
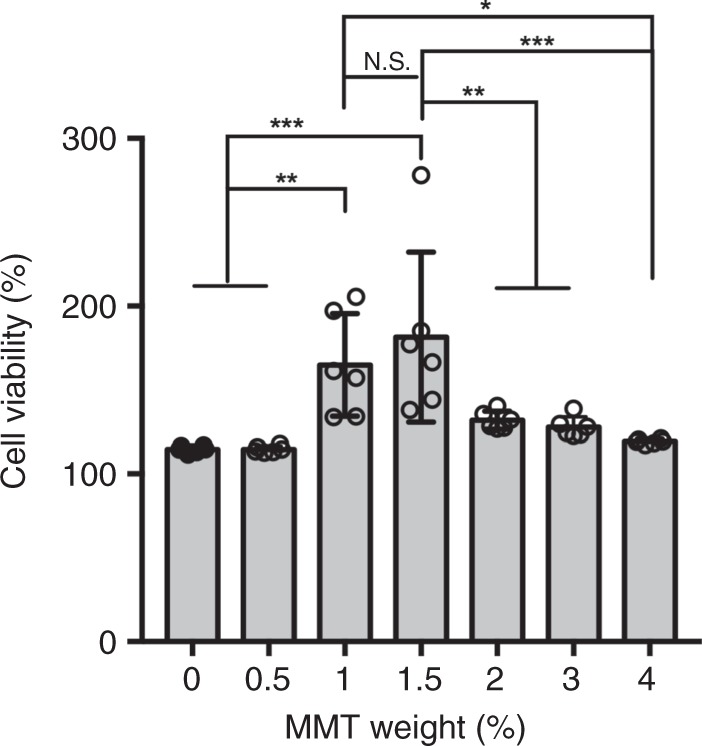
Assessment of cell cytotoxicity. The viability of encapsulated cells in photocross-linked MeGC-MMT nanocomposite hydrogels with various amount of MMT ranging from 0 to 4%. The hydrogels were cultured for 24 h. Error bars indicate standard deviation, *p < 0.05, **p < 0.01, ***p < 0.001, and NS, no significance (ANOVA followed by Tukey’s post hoc test)

**Fig. 4:**
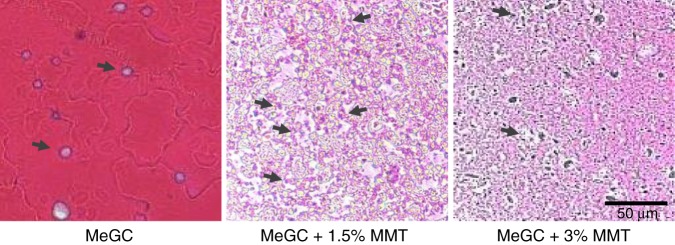
Hematoxyline and eosin (H&E) staining of encapsulated cells in photocross-linked MeGC-MMT nanocomposite hydrogels with various amount of MMT (0%, 1.5%, 3%). The hydrogels were cultured for 7 days (*n* = 3), scale bar = 50 μm, arrows indicate the cells

Interconnected porous microstructure was created in the MeGC-MMT nanocomposite hydrogels, the viability of cells inside which was further investigated to optimize the composition of MMT for bone tissue engineering. BMSCs were encapsulated inside of various MeGC-MMT nanocomposite hydrogels and MeGC hydrogels were employed as control. After 24-h culture, the cell viability of the 1% and 1.5% MeGC-MMT groups was significantly higher compared with the other groups (Fig. 3; Supplementary Fig. 3). Considering the initial characterization of the composite biomaterial, including the morphology, porosity, and cell viability, we chose the 1.5 and 3% MMT groups for further investigation. The hematoxyline and eosin (H&E) staining images confirm the interconnected microporous structure in the 1.5 and 3% MMT groups, and the highest number of cells was observed in the 1.5% MMT group, while MeGC hydrogel exhibited a smooth surface (Fig. 4). In addition, round-shaped cells were observed in the MeGC image, while spread cell morphology was seen in the MMT containing nanocomposite hydrogels. Taken together, the 1.5% MeGC-MMT nanocomposite hydrogel group presented the best support for cell proliferation.

### Bioactivity of MeGC-MMT nanocomposite hydrogels

**Fig. 5:**
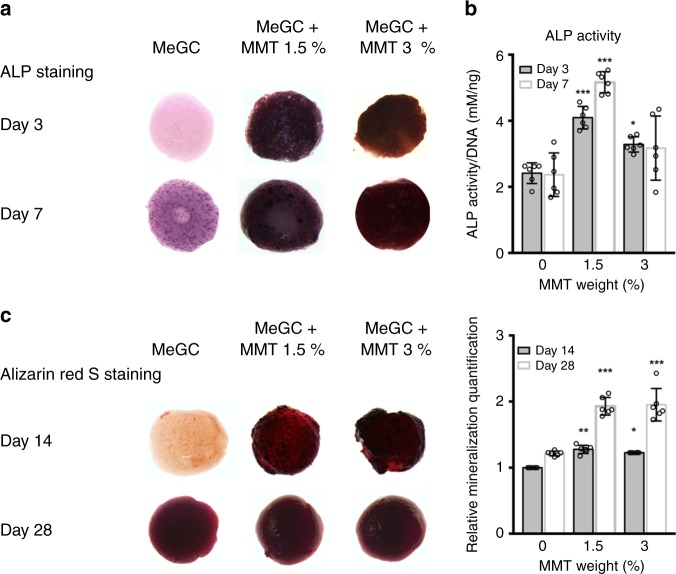
Osteoconductivity in vitro. The bioactivity of encapsulated cells in photocross-linked MeGC-MMT nanocomposite hydrogels with various amount of MMT (0%, 1.5%, 3%). The hydrogels were cultured in the osteogenic medium for various time. **a** ALP staining and **b** ALP activity were performed at days 3 and 7. **c** Alizarin red S staining was carried out at days 14 and 28, and their quantification was also evaluated. Error bars indicate standard deviation (three independent cultures, *n* = 6). *p *<* 0.05, **p < 0.01, and ***p < 0.001 compared with the control MeGC group with 0% MMT (ANOVA followed by Tukey’s post hoc test)

Differentiation of BMSCs to osteoblasts typically can be evaluated by the expression of early markers, such as ALP and the ultimate calcium deposition^29^. The BMSCs encapsulated in the MeGC-MMT (0%, 1.5 and 3%) were cultured for various duration. ALP staining and ALP activity at day 3, 7 and alizarin red S staining and its quantification at day 14, 28 were carried out (Fig. 5). The 1.5% MeGC-MMT group exhibited the most intensified staining of ALP at both days 3 and 7 compared with the other two groups (Fig. 5a), and ALP activity also showed the highest values for 1.5% MeGC-MMT group at both time points (Fig. 5b); while comparable mineralization to the 3% MeGC-MMT group (Fig. 5c).

**Fig. 6:**
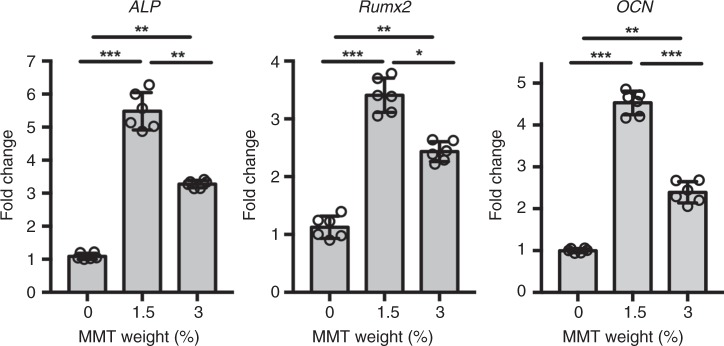
Gene markers evaluation of osteogenesis. The bioactivity of encapsulated cells was evaluated with qRT-PCR in photocross-linked MeGC-MMT nanocomposite hydrogels with various amount of MMT (0%, 1.5%, 3%). The hydrogels were cultured in the osteogenic medium for various time. *ALP* and *Runx2* were examined at day 7, and *OCN* was measured at day 14. Error bars indicate standard deviation (three independent cultures, *n* = 6), *p < 0.05, **p < 0.01, and ***p < 0.001 (ANOVA followed by Tukey’s post hoc test)

qRT-PCR was employed to evaluate the differentiation of BMSCs encapsulated in the MeGC-MMT nanocomposite hydrogels at a gene level. The gene expression of *ALP*, an early osteogenic marker, and *Runx2*, one of the most specific osteogenic differentiation markers in the earlier stage and *OCN*, a late osteogenic marker, was examined at days 7 and 14, respectively, and these results are presented in Fig. 6. Consistent results confirmed that the 1.5% MeGC-MMT exhibited the most powerful ability to promote the differentiation of BMSCs with a 5.5-, 3.4-, and 4.5-fold increase of the gene expression of *ALP*, *Runx2*, and *OCN* compared with the pure MeGC group. For the 3% MeGC-MMT group, statistically significant elevation of all three gene expression was observed as well; however, compared with the 1.5% MMT group, the ability of osteogenesis was significantly limited.

### In vivo bone regeneration of MeGC-MMT nanocomposites

**Fig. 7:**
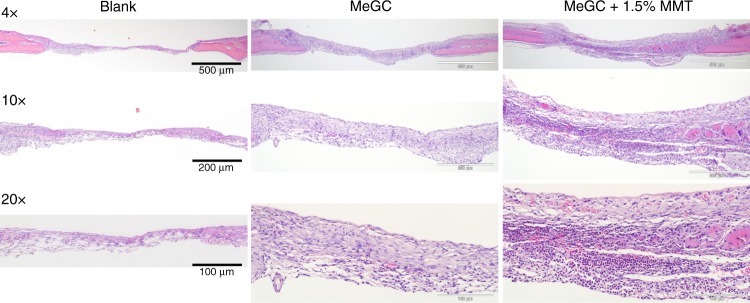
In vivo cell infiltration assessment. Histological analysis of native cell infiltration in the hydrogels with hematoxyline and eosin (H&E) staining in calvarial defects, 10 days post surgery

We took a further step to translate our 1.5% MeGC-MMT nanocomposite hydrogel to the in vivo critical-sized calvarial defect model of mice to evaluate bone regeneration. First, MeGC and 1.5% MeGC-MMT hydrogels were injected in the defects, and mice were euthanized 10 days post surgery to harvest the tissue along with remaining hydrogels inside the defects. Sections were stained with H&E in Fig. 7; overt native cell recruitment was observed. There was highly improved cell infiltration with thick granulation tissue formation composed of inflammatory cells and collagen-producing fibroblasts in the defect treated with MeGC-MMT nanocomposite hydrogel compared with the pure MeGC hydrogel group, while poor cell infiltration was observed in the blank control group.

**Fig. 8:**
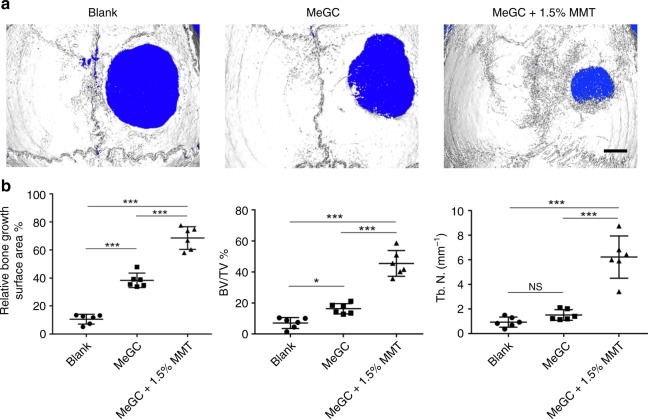
Evaluation of in vivo bone regeneration. **a** Microcomputed tomography images of calvarial defects treated with hydrogels or left empty, 6 weeks post surgery, scale bar = 1 mm. **b** μCT quantification of bone regeneration in calvarial defects. Relative bone growth surface area, bone volume density (BV/TV%), and the trabecular number (Tb.N., mm^−1^). *p < 0.05, ***p < 0.001, and NS, no significance (ANOVA followed by Tukey’s post hoc test). Error bars indicate standard deviation (*n* = 6)

As time progressed to week 6 post surgery, all mice were euthanatized for tissue collection. Ex vivo high resolution μCT was employed to evaluate the status of bone healing. The size of the remaining defect treated with 1.5% MeGC-MMT nanocomposite hydrogel was remarkably smaller than that of the other two groups, representative images are shown in Fig. 8a. The relative new bone growth surface area, bone volume/tissue volume (BV/TV %), and the trabecular number (Tb. N., mm^−1^) were extracted from the μCT images. The normalization was based on the original 3 mm defect area (Fig. 8b). The defects treated with MeGC and 1.5% MeGC-MMT hydrogels were covered by new bone at 38 and 69%, respectively, whereas the defects left empty exhibited a minimal healing 6 weeks post surgery (10%). The BV/TV and Tb. N. rose up to 46% and 6.2 mm^−1^ for the 1.5% MeGC-MMT group, considerably higher compared with that of the MeGC group (16% and 1.5) or the blank group (7% and 0.9). The 1.5% MeGC-MMT group resulted in the most effective bone repair in the absence of any exogeneous growth factor or stem cells.

**Fig. 9:**
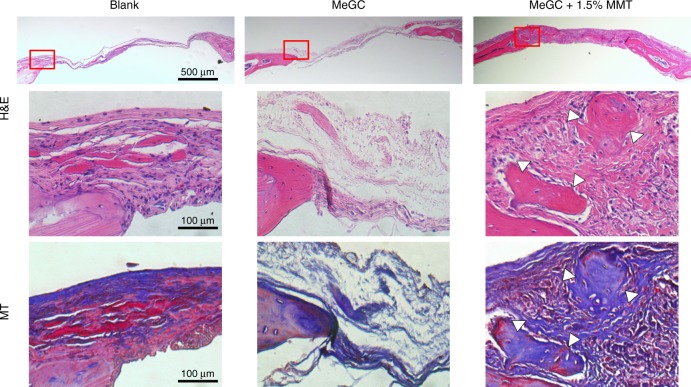
Histological analysis of bone regeneration in calvarial defects, 6 weeks post surgery. Hematoxyline and eosin (H&E) staining (scale bar = 500 μm), and magnified images of H&E and Masson trichrome staining (red boxes represent the magnified areas, scale bar = 100 μm; arrowheads indicate the new bone formation)

The quality of new bone formation was further examined by histological evaluation with H&E, Masson trichrome staining (Fig. 9). The defect treated with 1.5% MeGC-MMT nanocomposite hydrogel was occupied with newly formed bone, and thick soft tissue and bone-like tissue connected the edges, 6 weeks post surgery. The blank and MeGC groups presented very little bone tissue only on the edges of the defects and very thin soft tissue connecting the defects. Masson trichrome staining revealed an osteoid matrix formed on the edge of defects treated with the 1.5% MeGC-MMT nanocomposite hydrogel, whereas defects without any treatment or treated with the MeGC hydrogel were only filled with fibrous soft tissue with minimal bone healing. We have also performed immunohistochemical staining for the osteogenic markers Runx2 and OCN (Supplementary Fig. 8). Strong staining was observed in the 1.5% MeGC-MMT nanocomposite hydrogel group presenting osteoblastic cells, while weak immunostaining for Runx2 and OCN was detected within the fibrous tissue induced with the blank and MeGC groups. No signs of inflammatory responses were noted in all treated groups.

## Discussion

Injectable formulations of living cells and bioactive molecules using hydrogels would be an ideal route of administration to the target area without any surgical incisions. In particular, high porosity and pore interconnectivity are critical for cell ingrowth and mass transport in 3D constructs^27^. However, current technology of preparing such interconnected microporous hydrogels by using sacrificing porogen requires severe porogen removal steps employing acid or base, high temperature, solvent that may have significant toxicity to encapsulating cells. This study demonstrated a proof-of-concept of creating interconnected microporous structure in in situ-forming hydrogels by exploring intercalation chemistry in polymer chains with nanoclay to facilitate cell filtration, adhesion, proliferation, and differentiation for tissue engineering^27^. The positively charged MeGC polymers were well-mixed with weakly negatively charged MMT. The delamination of the individual layers of MMT evenly distributed inside the cured nanocomposite hydrogel, probably led to the interconnected microporous structure. The unique structure of smectites contributes to the microstructure change through interactions between nanoclay and polymer chains creating interlay-pores and inter-particle spaces. This discovery is of considerable importance in the development of the next generation of in situ-forming hydrogels with high porosity, because the process of tissue formation is a highly orchestrated set of temporal and spatial events that involve infiltration and proliferation of the stem/progenitor cells, matrix deposition, and vascularization.

In recent years, our group has developed a series of chitosan-based injectable hydrogels, which were designed for bone tissue engineering. For example, Arg–Gly–Asp (RGD) and phosphoserine (PS)-conjugated MeGC (PS–RGD–MeGC) hydrogel was synthesized to improve cell adhesion by the RGD motifs and promote osteogenesis by enhancing cell–matrix interactions and hydroxyapatite nucleation through PS^30^. Polysulfonate MeGC was rationally designed to sequester and stabilize endogenous bone morphogenic protein-2 (BMP-2), therefore to achieve osteogenesis without applying its exogenous supraphysiological dosage^31^. In addition, the bone-forming sterosomes^32^, the small molecular drug, i.e. phenamil, and siRNA knocking down noggin expression^33,34^ were embedded in MeGC hydrogels to enhance bone regeneration. All the efforts were proven to be effective in vivo in the calvarial defects of mice to a large extent, however, none of the formulation alone has achieved fully satisfied results within the experimental time yet, typically 6 weeks. One of the phenomena in all those injectable chitosan-based hydrogels is that they all bear a continuous microstructure without any pores (Figs. 2, 4), which probably limits their osteoconductive capability. The current MeGC-MMT formulation significantly promoted bone regeneration without any growth factor, small molecular drug, or gene.

Chitosan, a naturally occurring polymer, is appealing for tissue-engineering applications due to its high biocompatibility and hydrophilicity. MeGC hydrogels can be prepared under mild conditions with visible blue light. The MeGC hydrogel system supported proliferation and extracellular matrix deposition of encapsulated mesenchymal stem cells as well^25,26,32,35^. MMT belongs to the smectites with a unique structure and high aspect ratio, no harm to the environment or cells (Fig. 3) and low cost. Typically, the mechanical properties of hydrogels restrict their application on hard tissue engineering, such as bone. The literature reported that the inclusion of nanoclay in the polymer matrices significantly improve the mechanical and thermal properties^36–38^. Young’s modulus and EWC (Fig. 1b, c) were analyzed and, with the increment of MMT, the mechanical properties were remarkably improved. The Young’s modulus was increased more than six times with the 4% MeGC-MMT nanocomposite hydrogel compared with that of MeGC hydrogel (<10 kPa), and it was around 30 kPa for the 1.5% MeGC-MMT nanocomposite hydrogel.

MMT has been used as a filler in hard scaffolds, such as chitosan–MMT–hydroxyapatite^24^, gelatin–MMT–cellulose^23^ scaffolds and silk biomaterials^22^ for bone tissue formation. To the best of our knowledge, it is the first report to utilize MMT to create interconnected microporous structure in chitosan-based in situ-forming hydrogel just simply by intercalation chemistry (Supplementary Fig. 4). We confirmed the existence of pores in the wet state of hydrogel with low-vacuum SEM (Fig. 2) and also with frozen sections (Fig. 4) to avoid the ambiguity as the evaporation of water in the hydrogel leading to the formation of pores.

Further in vitro characterization of the combination of MeGC and MMT showed that the optimal weight of MMT was 1.5% w/v, to exhibit the strongest conductive ability in BMSCs, confirmed with ALP and mineralization staining and their corresponding quantification (Fig. 5), as well as specific gene expression (Fig. 6). We tempted to culture BMSCs only in the basal medium in the MeGC-MMT hydrogels, and osteogenic markers (*ALP* and *Runx2*) were upregulated on day 7 (Supplementary Fig. 6), indicating potential osteoinductive ability of this composite. Further experiments of ectopic bone formation should be carried out to confirm this speculation. The most appealing character of this nanocomposite hydrogel is their ability in vivo to recruit native cells for de novo bone formation (Fig. 7). Satisfactory results were obtained in the mouse calvarial defect model only by applying the material itself in the absence of cells or growth factors. This feature is of importance for translation to the clinical application, as cells were not necessary to be harvested and re-implanted back to the patients or expensive growth factors, such as BMP-2, to be applied. In addition, we have treated the mouse calvarial defect with commercially available collagen sponge^39^ and demineralized bone matrix (DBX)^40^ for 6 weeks as positive controls (Supplementary Fig. 7). The defects treated with 1.5% MeGC-MMT hydrogels, collagen sponge, and DBX were covered by new bone at 69, 23, and 26%, respectively. The BV/TV and Tb. N. rose up to 46% and 6.2 mm^−1^ for the 1.5% MeGC-MMT group, considerably higher compared with that of the collagen sponge group (6.4% and 2.7) or the DBX group (13.1% and 4.3). Compared with the commercially available materials, our newly developed MeGC-MMT hydrogel presents great potential for bone healing.

The surface charges and the exchange ability of MMT were well studied for delivery purpose^11^. Taking advantage of this, further development of this chitosan-based hydrogel system specifically for bone tissue engineering is indispensable. The combination and optimization of PS–RGD–MeGC or polysulfonate MeGC and MMT hold great promise for bone regeneration. The critical defect fully filled with regenerated bone within experimental time will soon be achieved. The underlying mechanism should be further studied to elucidate the strong osteoconductivity of this biomaterial and the type of tissue that replaces the hydrogel site with time progression needs to be closely monitored as well in our future work.

Our novel nanocomposite hydrogel system can be utilized not only as 3D scaffolds for non-load-bearing injury site such as cranial defects but also synthetic biological carriers for stem cells, bioactive agents, or currently available tissue grafts for regenerative medicine. We did not evaluate mechanical properties of the regenerated bone in this study, because the calvarial defect is small and the healing site is not load bearing. However, the further study of biomechanical properties will be needed in other fracture models such as large segmental defects. It is possible that the hydrogel may not possess sufficient mechanical properties in the load-bearing bone defect sites. A promising alternative is to combine the hydrogel with solid particles or bone graft materials.

In conclusion, we have demonstrated that with the help of intercalation chemistry, proportional MMT mixing with MeGC leads to the formation of interconnected microporous structure, which can promote native cell infiltration, proliferation, and in situ differentiation in the absence of any growth factors, small molecular drugs, or genes. This biocompatible, bioactive, and injectable nanocomposite material shows great promise being applied in a wide range of tissue regeneration.

## Methods

### Materials

Glycol chitosan (GC, ~100 kDa, 072-1581) was purchased from Wako Chemicals USA, Inc. (Richmond, VA, USA). MMT (682659-500G), glycidyl methacrylate (GMA, 151238-100G), 1-ethyl-3-(3-dimethylaminopropyl)-carbodiimide (EDC, E1769-10G), tween-20 (p1379-500ML), *p*-nitrophenyl phosphate (N7653-100ML), β-glycerophosphate (G5422-100G), _L_-ascorbic acid (A5960-25G), dexamethasone (D4902-25MG), Nitro Blue Tetrazolium (NBT, N5514-25TAB), 5-bromo-4-chloro-3-indoxylphosphate (BCIP, B6149-50MG), alizarin red S (A5533), and ethylenediaminetetraacetic acid (EDTA, EDS-1KG) were supplied by Sigma-Aldrich (St. Louis, MO, USA). The mouse bone marrow stromal cell line (BMSCs, D1 ORL UVA [D1], D1 cell, CRL-12424) was obtained from American Type Culture Collection (ATCC, Manassas, VA, USA). High glucose Dulbecco’s Modified Eagle’s Medium (DMEM, 11995-065), penicillin/streptomycin (100 U/mL, 15140122) were purchased from Life Technologies (Grand Island, NY, USA), and fetal bovine serum (FBS, MT35010CV) was supplied by Mediatech Inc. (Manassas, VA, USA). The live/dead staining solution (L3224) and cDNA transcription kit (18080-051) were purchased from Invitrogen (Carlsbad, CA, USA). Trizol reagent (15596018) and RNeasy Mini Plant Kit (74106) were supplied by Qiagen (Valencia, CA, USA). Pierce BCA Protein Assay Kit was obtained from Thermo Scientific (23235, Rockford, IL, USA). The nude mice were purchased from Charles River Laboratories (Wilmington, MA, USA). All solvents and products were used as received.

### Preparation of MeGC and MeGC-MMT nanocomposite hydrogels

In all, 2% (w/v) GC and GMA were mixed at 1:1 molar ratio of GMA to the amino groups in GC in Milli-Q water. The solution was adjusted to pH 9.0, and placed on a shaker at room temperature. After 40 h, pH was readjusted to 7.0, and the solution was dialyzed with 50 kDa tubes against Milli-Q water for 16 h. After lyophilization and rehydration with phosphate-buffered saline (PBS), a 4% (w/v) MeGC solution was obtained. MMT was dispersed in Milli-Q water and yield 100 mg mL^−1^ stock dispersion. Various amount of MMT (w/v) was mixed with 4% (w/v) MeGC solution, and the hydrogels were cured under visible blue light with riboflavin as the photoinitiator (final concentration 6 µM). The final concentration of MeGC is 2% (w/v) with various amount of MMT.

### Characterization of MeGC-MMT nanocomposite hydrogels

A 400 μL of hydrogel mixture was cured for 80 s in a 48-well plate, and compressive modulus was measured using a flat-ended indenter (1.6 mm in diameter) on an Instron Electro-Mechanical Testing Machins (Instron, Model 5564, Norwood, MA, USA). The Young’s modulus was determined from the slope of linear portion of the obtained stress-strain curve using a Poissons’ ratio of 0.25^25,41,42^.

Hydrogels were equilibrated in PBS for 24 h and lyophilized for 16 h to obtain dry gels. EWC was calculated using the following equation

1 $${\mathrm{EWC}} = \frac{{M_w - M_d}}{{M_w}},$$

where *M*_w_ and *M*_d_ refer to the weight of wet and dry hydrogels, respectively.

The degradation of hydrogels was carried out for 42 days. Hydrogels were incubated at 37 °C in cell growth medium (DMEM, 10% FBS, and 1% P/S), which was replaced every 7 days. At a pre-set time, hydrogels were lyophilized for weight measurement. The present residual weight of hydrogels was calculated using the following equation

2 $${\mathrm{Residual}}\;{\mathrm{dry}}\;{\mathrm{gel}}\;{\mathrm{weight}} \% = \frac{{M_{t}}}{{M_{0}}} \times 100{\mathrm{\% }},$$

where *M*_0_ and *M*_t_ refer to the weight of hydrogels at time 0 (hydrogels did not undergo degradation) and *t*, respectively.

Hydrogels were imaged in low vacuum using scanning electron microscopy with X-ray microanalysis (SEM/EDS, FEI Nova NanoSEM 230, Hillsboro, OR, USA) to observe the microstructure and chemical compositions. For Transmission Electron Microscope (TEM) analysis, hydrogels were embedded in sucrose-PVP and mounted on Cu EM grid after sectioning with glass 45^o^ knife with 100 -nm thickness (Leica UC6/F6 −100 ^o^C), then imaged with JEM1200EX. The thermogravimetric analysis (TGA) was performed with the lyophilized hydrogels from 50 ^o^C to 600 ^o^C at 30 ^o^C min^−1^ speed. The Powder X-ray Diffraction (XRD, Bruker Corporation, Germany) pattern was collected from 2 to 10^o^ (2*θ* range) with a diffractometer using Ni-filtered CuKα X-ray radiation (*λ* = 1.5418 Å).

### 3D cell culture in MeGC-MMT nanocomposite hydrogels

BMSCs at a density of 2 × 10^6^ cells mL^−1^ were mixed in MeGC-MMT dispersion. The hydrogel was cured by exposing 40 μL of the suspension to visible blue light (400–500 nm, 500–600 mW cm^−2^, Bisco Inc., Schaumburg, IL) in the presence of riboflavin as a photoinitiator, (final concentration 6 µM). The resulting hydrogels were incubated in 1 mL of media accordingly.

### Cytotoxicity

Cytotoxicity of MMT in nanocomposite hydrogels was evaluated using alamar blue assay and live/dead staining (Invitrogen, Carlsbad, CA). Hydrogels with encapsulated BMSCs and MMT in various concentrations were incubated at 37 ^o^C and 5% CO_2_ in fresh growth medium. Then the medium was replaced with 10% (v/v) alamar blue solution in growth medium at a predetermined time. After a 3-h incubation, the fluorescence intensity (F) of alamar blue was measured at 585 nm with an excitation wavelength of 570 nm. For the blank group, the 10% (v/v) alamar blue solution was added in an empty well and incubated together with other samples. The relative cell viability (%) was calculated using the following equation

3 $${\mathrm{Relative}}\;{\mathrm{cell}}\;{\mathrm{viability}} = \frac{{F_{s} - F_{b}}}{{F_{c} - F_{b}}} \times 100{\mathrm{\% ,}}$$

where *F*_s_, *F*_c_, and *F*_b_ refer to the fluorescence intensity of the sample after incubation for 24 h, intensity of the corresponding sample without treatment, and the blank, respectively.

### ALP and alizarin red S staining and quantification

Gels were incubated in osteogenic media (growth medium was supplemented with 10 mM β-glycerophosphate, 50 μg mL^−1^ l-ascorbic acid, and 100 nM dexamethasone). At a predetermined time, gels were fixed in 10% formalin for 20 min, rinsed with PBS, and incubated in a solution consisting of NBT and BCIP stock solutions in an ALP buffer (100 mM Tris, 50 mM MgCl_2_, 100 mM NaCl, pH 8.5) for 2 h, at room temperature. The stained samples were observed with an Olympus SZX16 Stereomicroscope (Olympus, Tokyo, Japan). ALP expression appeared in blue. For the ALP activity assay, gels were rinsed with PBS, incubated in a lysis buffer (0.1% Tween-20 in PBS) at 4 °C for 5 min. ALP activity was determined colorimetrically using *p*-nitrophenyl phosphate as a substrate and measured at 405 nm. The ALP activity was normalized to total DNA contents measured with the picogreen assay (Thermo Scientific, Rockford, IL).

Gels were fixed in 10% formalin for 20 min, rinsed with PBS, and incubated in 2% alizarin red S solution for 5 min at room temperature. Then, the gels were washed with PBS under gentle shaking for 16 h, and the PBS was changed at least three times. The stained samples were observed with the Olympus SZX16 Stereomicroscope. Calcium deposition appeared in red. The semi quantification of alizarin red S staining was carried out with acetic acid extraction and neutralization with ammonium hydroxide, followed by a colorimetric detection at 405 nm.

### RNA extraction and quantitative real-time PCR

The total RNA was extracted using Trizol reagent and RNeasy Mini kit. In all, 500 ng of total RNA was reversely transcribed to cDNA using a cDNA transcription kit (Invitrogen). Quantitative real-time PCR was performed with a LightCycler 480 PCR (Indianapolis, IN) with a 20 μL SYBR Green reaction system. PCR amplification was performed for 50 cycles. The expression of housekeeping gene (*GAPDH*) was employed to normalize gene expression levels. The primer sequences are listed in Supplementary Table 2.

### Calvarial defect model

All animal experiments were performed in accordance with the guidelines of the Chancellor’s Animal Research Committee at the University of California, Los Angeles. Calvaria of male CD-1 nude mice (8–12 weeks old) were exposed to a trephine drill under constant irrigation, and 3-mm full-thickness craniotomy defects were created in the right side of parietal bone with care to avoid injury to the underlying dura mater. Each defect was syringed with sterile saline solution to remove bone debris and then implanted with MeGC-MMT nanocomposite hydrogels through injecting MeGC-MMT sol followed by in situ curing with visible blue light or left without any treatment. After surgery, all animals were allowed to recover on a warm sheet and then transferred to the vivarium for postoperative care. In preparation for the operative treatment, all animals received analgesia with subcutaneous injections of buprenorphine at a concentration of 0.1 mg kg^−1^ for 3 days. To prevent a potential infection, all animals received drinking water including trimethoprim–sulfamethoxazole for 7 days.

### Micro-computerized tomography (μCT) scanning

Six weeks post implantation, animals were euthanized and calvarial tissues were harvested for analysis. The extracted calvarial tissues were fixed in 4% formaldehyde at room temperature with gentle shaking. After 48 h, the fixed samples were rinsed with PBS and imaged using the high-resolution μCT (SkyScan 1172; SkyScan, Kontich, Belgium) with 57 kVp, 184 μA, 0.5 mm aluminum filtration, and 10 μm resolution. Visualization and reconstruction of the data were obtained using the OsiriX MD imaging software. The new bone surface area was measured using ImageJ software (NIH, Bethesda, MD), and normalized to the original defect surface area (3 mm in diameter). Bone volume density (bone volume/tissue volume, BV/TV [%]) and trabecular number (TN; mm^−1^) were measured with CTan (Skyscan).

### Histological evaluation

MeGC-MMT hydrogels were frozen-sectioned to 5-μm-thick slides to evaluate the microstructure. The fixed tissues were decalcified in 10% EDTA solution under gentle shaking for 1 week. The EDTA solution was changed once at day 3. Decalcified samples were embedded in paraffin, and cut into 5-μm-thick sections. The tissue sections were deparaffinized and stained with H&E. Masson trichrome staining was also performed to detect new bone formation. The blue color, indicative of new or mature bone, was observed using an Olympus IX71 microscope. Additional sections underwent immunohistochemical analysis. The deparaffinized sections were processed with citric acid for antigen retrieval and thereafter incubated with the primary antibody osteocalcin (OCN, 1:150 dilution, Santa Cruz FL-95) and Runx2 (1:150 dilution, Santa Cruz F-2) and were detected by the HRP/DAB kit (Abcam). The sections were further counterstained with Mayer’s hematoxylin.

### Statistical analysis

Three independent experiments at least, unless otherwise stated, were performed and data were presented as mean ± standard deviation. Multiple comparisons were assessed using one-way or two-way analysis of variance (ANOVA). The analysis of variances followed by Tukey’s post hoc test was employed in this work and *p* < 0.05 was considered statistically significant.

### Reporting summary

Further information on research design is available in the Nature Research Reporting Summary linked to this article.

## Supplementary information

Supplementary Information

Reporting Summary

Source Data

## Data Availability

All relevant data are available within the article and Supplementary Information. The source data underlying Figs. 1B–D, 2, 3, 5B–C, and 66 and Supplementary Figs. 1, 4, 5, 6, and 7B are provided as a Source Data file. Requests for other materials should be addressed to the corresponding author.
