# Supplementary material for: Microporous methacrylated glycol chitosan-montmorillonite nanocomposite hydrogel for bone tissue engineering
Source: Nat Commun. 2019 Aug 6;10:3523. doi: 10.1038/s41467-019-11511-3 (PMC6684526; doi:10.1038/s41467-019-11511-3)
Supplement: Supplementary file 1 — Supplementary Information [file 41467_2019_11511_MOESM1_ESM.pdf]

Microporous Methacrylated Glycol Chitosan-Montmorillonite Nanocomposite Hydrogel for Bone Tissue Engineering

Cui and Kim et al.

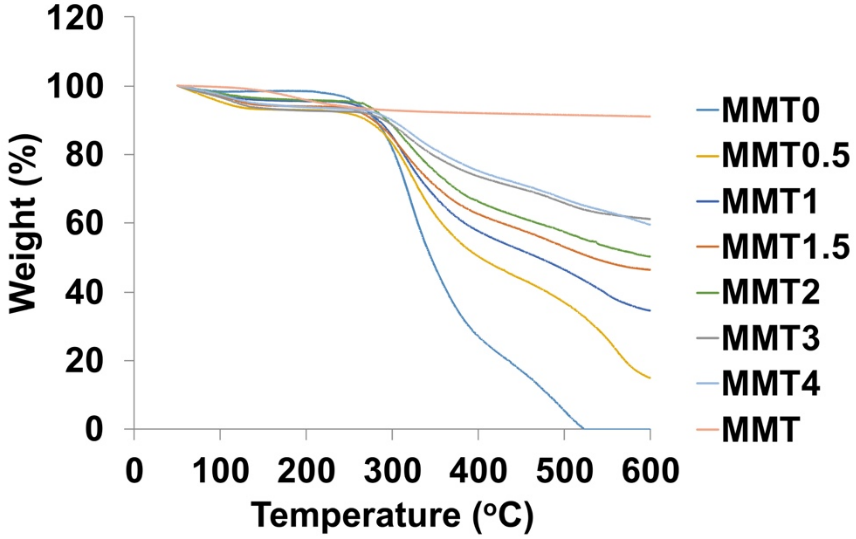

Supplementary Figure 1. TGA profiles of MMT, MeGC (MMT0) and various MeGC-MMT composites with 0.5-4% MMT.

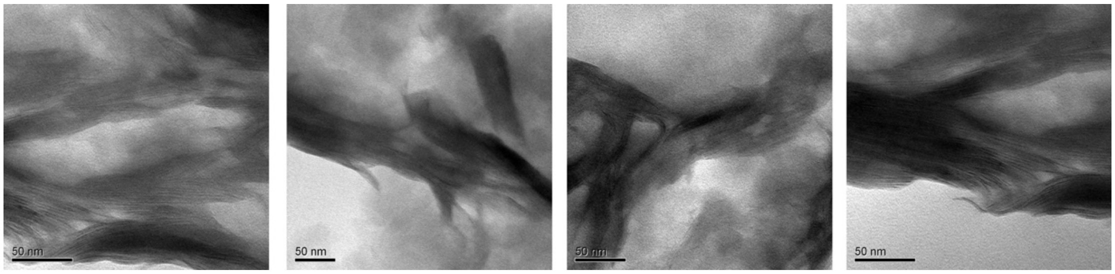

Supplementary Figure 2. TEM images of 1.5% MeGC-MMT composite biomaterial. (Scale bars = 50 nm)

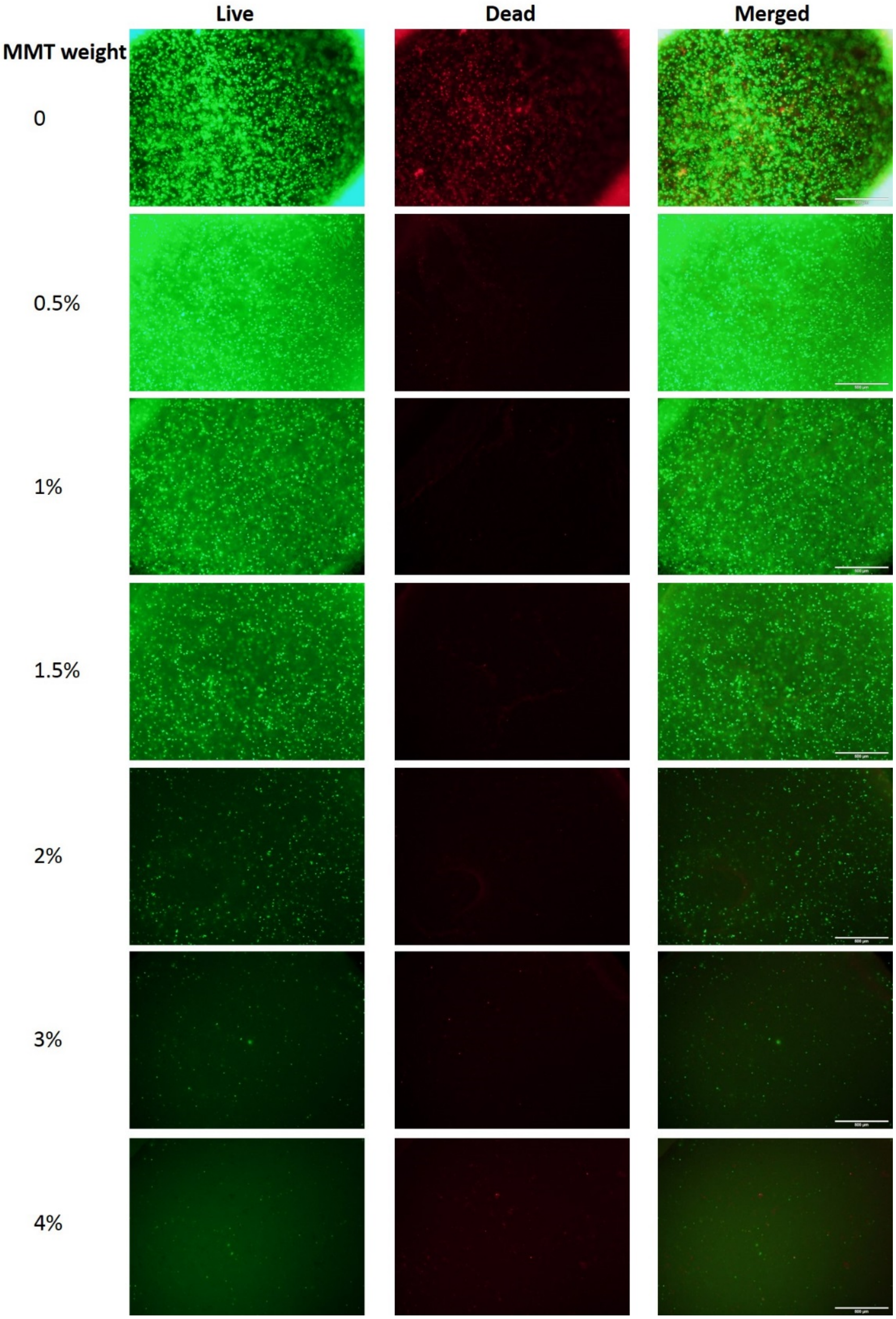

Supplementary Figure 3. The viability of encapsulated cells in photocrosslinked MeGC-MMT nanocomposite hydrogels with various amount of MMT ranging from 0% to 4%, characterized with Live/Dead staining. The hydrogels were cultured for 24 h (n = 3). Scale bars = 500 μm.

When the MMT mount exceeds 1.5%, the MeGC-MMT nanocomposite hydrogels become opaque. Therefore, the observation of cells with fluorescent microscope was obstructed. Nonetheless, the trend of cell viability is still obvious.

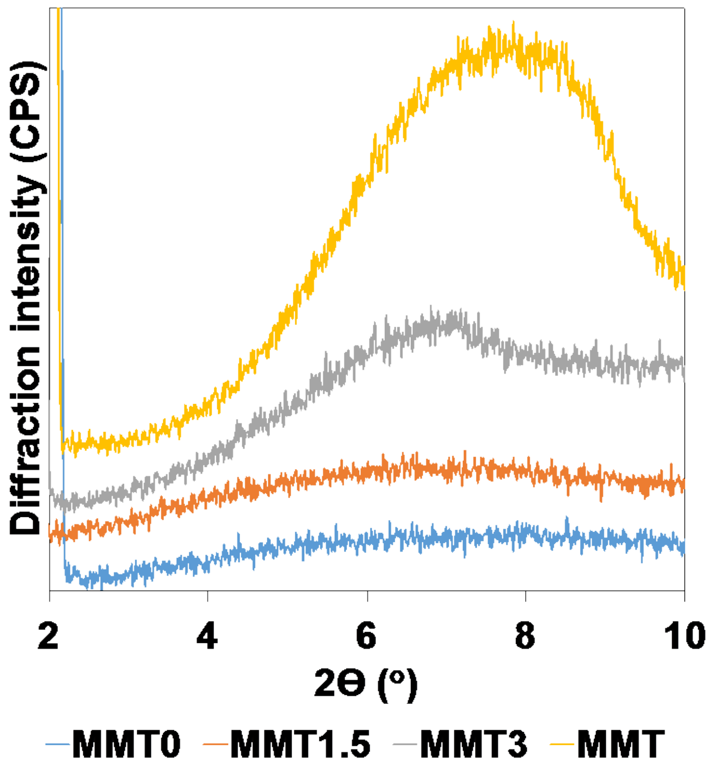

Supplementary Figure 4. XRD patterns of MMT only, MeGC (MMT0), 1.5% MeGC-MMT (MMT1.5) and 3% MeGC-MMT (MMT3) groups.

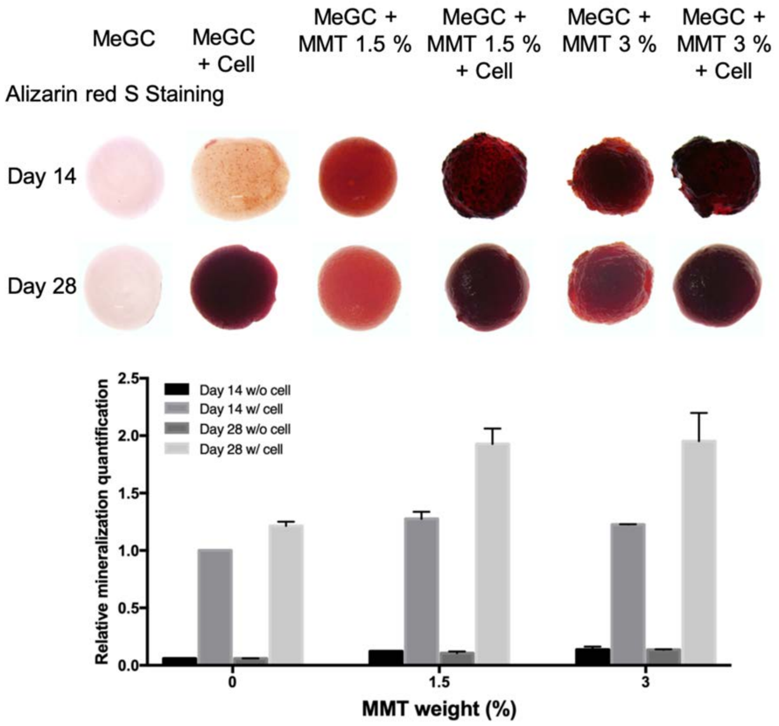

Supplementary Figure 5. Background alizarin red S staining of MeGC-MMT nanocomposite hydrogel (0%, 1.5%, 3%). Relative colorimetric quantification of alizarin red S staining was compared between

cell and no cell groups.

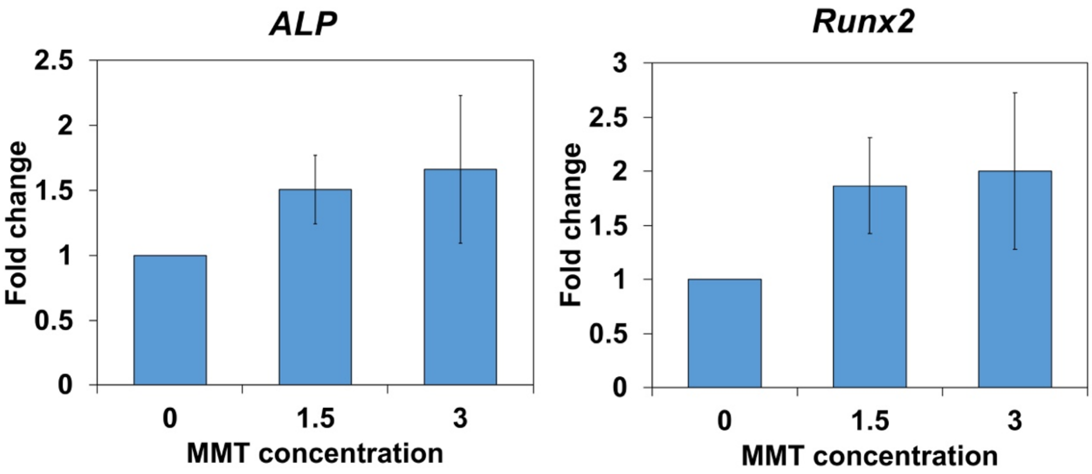

Supplementary Figure 6. The bioactivity of encapsulated cells evaluated with qRT-PCR in photocrosslinked MeGC-MMT nanocomposite hydrogels with various amount of MMT (0%, 1.5%, 3%). The hydrogels were cultured in growth medium. *ALP* and *Runx2* were examined at day 7.

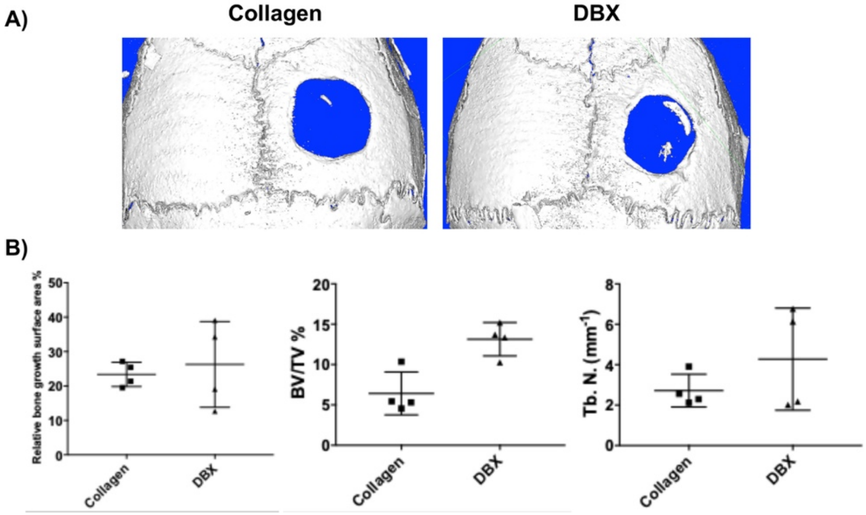

Supplementary Figure 7. (A) Microcomputed tomography images of calvarial defects treated with collagen sponge or demineralized bone matrix (DBX), 6 weeks post-surgery. (B) μCT quantification of bone regeneration in calvarial defects. Relative bone growth surface area, bone volume density (BV/TV%), and trabecular number (Tb.N., mm^{-1}), (n = 4).

Collagen sponge (Absorbable Collagen Sponges, 7510900) and DBX Putty (DePuy Synthes, MTF Biologics, 038010) were used for *in vivo* mouse study.

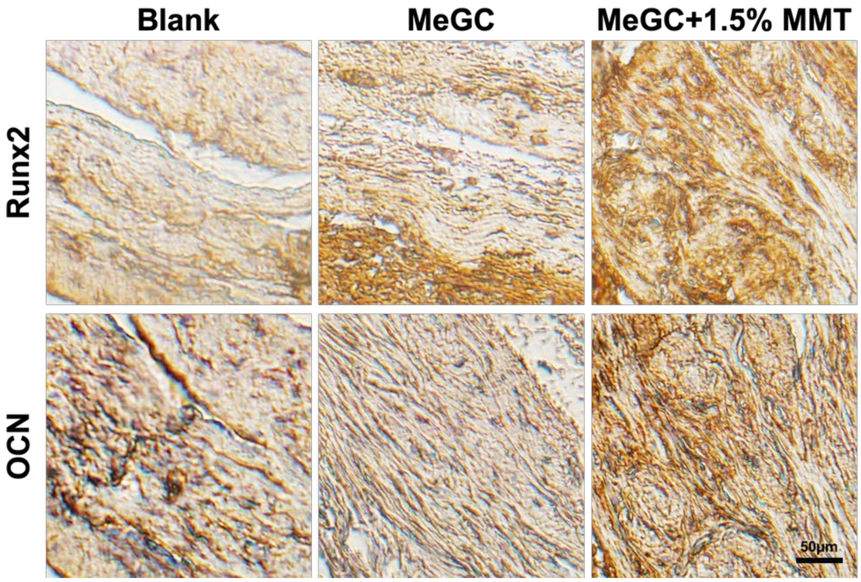

Supplementary Figure 8. Immunohistochemistry staining (Runx2 and OCN) of bone regeneration in calvarial defects, 6 weeks post-surgery, scale bar = 50 μm.

Supplementary Table 1. EDS analysis of MeGC-MMT composite biomaterial.

Atom%	MMT0	MMT0.5	MMT1	MMT1.5	MMT2	MMT3	MMT4
C K	57.29	50.99	49.67	53.16	48.26	39.81	39.34
N K	7.7						
O K	33.13	40.82	39.5	37.78	40.71	44.85	47.62
F K				0.29	1.87	0.23	0.79
Na K	0.76		0.79	0.52		0.79	0.45
Mg K		0.6	0.7	0.54	0.59	0.96	0.74
Al K		1.78	2.05	1.75	2.02	3.23	2.75
Si K	0.38	5.03	6.11	5	4.82	9.36	7.5
Ca K			0.19	0.14			
Cl K	0.75						
Ni L					0.76		
Au M		0.77	0.99	0.82	0.96	0.77	0.82

Supplementary Table 2. List of primer sequences

Genes	Sequence
GAPDH	AGGTCGGTGTGAACGGATTTG (forward)
	TGTAGACCATGTAGTTGAGGTCA (reverse)
Alkaline phosphatase (ALP)	GTTGCCAAGCTGGGAAGAACAC (forward)
	CCCACCCCGCTATTCCAAAC (reverse)
Runt-related transcription factor 2 (Runx2)	CGGTCTCCTTCCAGGATGGT (forward)
	GCTTCCGTCAGCGTCAACA (reverse)
Osteocalcin (OCN)	GGGAGACAACAGGGAGGAAAC (forward)
	CAGGCTTCCTGCCAGTACCT (reverse)
