# Supplementary material for: Microporous methacrylated glycol chitosan-montmorillonite nanocomposite hydrogel for bone tissue engineering
Source: Nat Commun. 2019 Aug 6;10:3523. doi: 10.1038/s41467-019-11511-3 (PMC6684526; doi:10.1038/s41467-019-11511-3)
Supplement: Supplementary file 2 — Reporting Summary [file 41467_2019_11511_MOESM2_ESM.pdf]

nature research

Corresponding author(s): Min Lee, Zhong-Kai Cui
Last updated by author(s): Jun 12, 2019

Reporting Summary

Nature Research wishes to improve the reproducibility of the work that we publish. This form provides structure for consistency and transparency in reporting. For further information on Nature Research policies, see Authors & Referees and the Editorial Policy Checklist.

Statistics

For all statistical analyses, confirm that the following items are present in the figure legend, table legend, main text, or Methods section.

n/a	Confirmed	
☐	☒	The exact sample size (*n*) for each experimental group/condition, given as a discrete number and unit of measurement
☐	☒	A statement on whether measurements were taken from distinct samples or whether the same sample was measured repeatedly
☐	☒	The statistical test(s) used AND whether they are one- or two-sided<br>*Only common tests should be described solely by name; describe more complex techniques in the Methods section.*
☒	☐	A description of all covariates tested
☐	☒	A description of any assumptions or corrections, such as tests of normality and adjustment for multiple comparisons
☐	☒	A full description of the statistical parameters including central tendency (e.g. means) or other basic estimates (e.g. regression coefficient) AND variation (e.g. standard deviation) or associated estimates of uncertainty (e.g. confidence intervals)
☐	☒	For null hypothesis testing, the test statistic (e.g. *F*, *t*, *r*) with confidence intervals, effect sizes, degrees of freedom and *P* value noted<br>*Give P values as exact values whenever suitable.*
☒	☐	For Bayesian analysis, information on the choice of priors and Markov chain Monte Carlo settings
☒	☐	For hierarchical and complex designs, identification of the appropriate level for tests and full reporting of outcomes
☒	☐	Estimates of effect sizes (e.g. Cohen's *d*, Pearson's *r*), indicating how they were calculated

Our web collection on statistics for biologists contains articles on many of the points above.

Software and code

Policy information about availability of computer code

Data collection	Full detail of softwares used for data collection can be found in the Methods section.
Data analysis	Full detail of softwares used for data analysis can be found in the Methods section.

For manuscripts utilizing custom algorithms or software that are central to the research but not yet described in published literature, software must be made available to editors/reviewers. We strongly encourage code deposition in a community repository (e.g. GitHub). See the Nature Research guidelines for submitting code & software for further information.

Data

Policy information about availability of data

All manuscripts must include a data availability statement. This statement should provide the following information, where applicable:

- Accession codes, unique identifiers, or web links for publicly available datasets
- A list of figures that have associated raw data
- A description of any restrictions on data availability

All relevant data are available within the manuscript and supplementary information. The source data underlying Figs 1B-D, 2, 3, 5 and 6 and Supplementary Figs 1, 4, 5 and 6B are provided as a Source Data file. Requests for other materials should be addressed to the corresponding author.

Field-specific reporting

Please select the one below that is the best fit for your research. If you are not sure, read the appropriate sections before making your selection.

☒ Life sciences ☐ Behavioural & social sciences ☐ Ecological, evolutionary & environmental sciences

Life sciences study design

All studies must disclose on these points even when the disclosure is negative.

Sample size	Sample size was chosen to ensure an adequate statistical power.
Data exclusions	No data were excluded from analysis.
Replication	Reproducibility of experimental findings was assessed by performing experiments with independent biological replicates separately collected. Experimental variation is reported in the applicable figures as standard error of the mean.
Randomization	Not relevant
Blinding	Investigators were blinded during data collection and analysis.

Reporting for specific materials, systems and methods

We require information from authors about some types of materials, experimental systems and methods used in many studies. Here, indicate whether each material, system or method listed is relevant to your study. If you are not sure if a list item applies to your research, read the appropriate section before selecting a response.

Materials & experimental systems

n/a	Involved in the study	
☐	☒	Antibodies
☐	☒	Eukaryotic cell lines
☒	☐	Palaeontology
☐	☒	Animals and other organisms
☒	☐	Human research participants
☒	☐	Clinical data

Methods

n/a	Involved in the study	
☒	☐	ChIP-seq
☒	☐	Flow cytometry
☒	☐	MRI-based neuroimaging

Antibodies

Antibodies used	Osteocalcin (OCN, 1:150 dilution, Santa Cruz FL-95) and Runx2 (1:150 dilution, Santa Cruz F-2) were used in our experiment.
Validation	Antibodies were validated by using a positive control.

Eukaryotic cell lines

Policy information about cell lines

Cell line source(s)	Mouse bone marrow stromal cells (D1 ORL, ATCC)
Authentication	Functional and molecular authentication are assessed using a panel of antibody and qRT-PCR markers.
Mycoplasma contamination	All cell lines tested negative for mycoplasma contamination.
Commonly misidentified lines (See ICLAC register)	No cell lines used in this study were found in the database of commonly misidentified cell lines that is maintained by ICLAC and NCBI Biosample.

Animals and other organisms

Policy information about studies involving animals; ARRIVE guidelines recommended for reporting animal research

Laboratory animals	Male CD-1 nude mice (8-12 weeks old)
Wild animals	N/A
Field-collected samples	N/A
Ethics oversight	All animal experiments were performed in accordance with the guidelines of the Chancellor's Animal Research Committee at the University of California, Los Angeles.

Note that full information on the approval of the study protocol must also be provided in the manuscript.
